# Targeting B Cell Maturation Antigen (BCMA) in Multiple Myeloma: Potential Uses of BCMA-Based Immunotherapy

**DOI:** 10.3389/fimmu.2018.01821

**Published:** 2018-08-10

**Authors:** Shih-Feng Cho, Kenneth C. Anderson, Yu-Tzu Tai

**Affiliations:** ^1^LeBow Institute for Myeloma Therapeutics and Jerome Lipper Multiple Myeloma Center, Dana-Farber Cancer Institute, Boston, MA, United States; ^2^Harvard Medical School, Boston, MA, United States; ^3^Division of Hematology and Oncology, Department of Internal Medicine, Kaohsiung Medical University Hospital, Kaohsiung Medical University, Kaohsiung, Taiwan; ^4^Faculty of Medicine, College of Medicine, Kaohsiung Medical University, Kaohsiung, Taiwan

**Keywords:** multiple myeloma, B-cell maturation antigen, targeted immunotherapy, monoclonal antibody, chimeric antigen receptor T cell, monoclonal antibody drug conjugate, bi-specific antibody

## Abstract

The approval of the first two monoclonal antibodies targeting CD38 (daratumumab) and SLAMF7 (elotuzumab) in late 2015 for treating relapsed and refractory multiple myeloma (RRMM) was a critical advance for immunotherapies for multiple myeloma (MM). Importantly, the outcome of patients continues to improve with the incorporation of this new class of agents with current MM therapies. However, both antigens are also expressed on other normal tissues including hematopoietic lineages and immune effector cells, which may limit their long-term clinical use. B cell maturation antigen (BCMA), a transmembrane glycoprotein in the tumor necrosis factor receptor superfamily 17 (TNFRSF17), is expressed at significantly higher levels in all patient MM cells but not on other normal tissues except normal plasma cells. Importantly, it is an antigen targeted by chimeric antigen receptor (CAR) T-cells, which have already shown significant clinical activities in patients with RRMM who have undergone at least three prior treatments, including a proteasome inhibitor and an immunomodulatory agent. Moreover, the first anti-BCMA antibody–drug conjugate also has achieved significant clinical responses in patients who failed at least three prior lines of therapy, including an anti-CD38 antibody, a proteasome inhibitor, and an immunomodulatory agent. Both BCMA targeting immunotherapies were granted breakthrough status for patients with RRMM by FDA in Nov 2017. Other promising BCMA-based immunotherapeutic macromolecules including bispecific T-cell engagers, bispecific molecules, bispecific or trispecific antibodies, as well as improved forms of next generation CAR T cells, also demonstrate high anti-MM activity in preclinical and even early clinical studies. Here, we focus on the biology of this promising MM target antigen and then highlight preclinical and clinical data of current BCMA-targeted immunotherapies with various mechanisms of action. These crucial studies will enhance selective anti-MM response, transform the treatment paradigm, and extend disease-free survival in MM.

## Introduction

Multiple myeloma (MM), the second most common hematologic malignancy in the United States, accounts for 1% of malignancies and 10% of hematologic cancers (1). This tumor is characterized by the expansion of malignant plasma cells (PCs) in the bone marrow (BM), associated with excessive production of monoclonal immunoglobulins in blood and urine in patients. In addition, MM patients develop significant osteolytic bone lesions and have immunodeficiency that compromises both longevity and quality of life (2, 3). For the past two decades, the clinical outcome of MM patients has shown remarkable improvements primarily due to the incorporation of novel therapeutic agents into conventional treatments. Specifically, the addition of proteasome inhibitors (PI) and immumomodulatory drugs (IMiDs) has significantly increased response rate, progression-free, and overall survival in both relapsed and newly diagnosed MM patients, compared with conventional therapies (4–7). The addition of monoclonal antibodies (MoAbs) elotuzumab and daratumumab as immunotherapies in MM has further improved patient outcome. The use of autologous stem cell transplantation also results in better outcome. However, MM remains incurable for most patients, since drug-resistant clones constantly emerge and evolve (8). Persistence of minimal residual disease (MRD) is often seen and patients with MRD-negativity also relapse. Particularly, the overall survival of patients with relapsed disease after PIs IMiDs, and MoAbs treatment is extremely low. Thus, more efficacious therapies and novel strategies are urgently needed if we are to develop curative therapies.

Multiple myeloma develops from a premalignant precursor condition monoclonal gammopathy of undetermined significance, progressing to smoldering MM, then active MM, majorities of which ultimately advancing to end-stage PC leukemia. Genetic and epigenetic processes are present initially and underlie this progression, including hyperdiploidy of chromosomes, translocation of immunoglobulin heavy chain, deregulation of cell cycle genes, alteration of NFκB pathways, and abnormal DNA methylation patterns (9–11). Besides complex molecular aberrations, MM cells are heavily dependent on their BM microenvironment to support their growth, survival, and the development of drug resistance. Tumor cells closely interact with BM accessory cells in bidirectional fashions *via* cell–cell contact and/or production of a variety of factors, which ultimately promotes MM cell expansion, while impairing immune surveillance and effector function against MM cells. These MM-supporting cells include BM stromal cells (BMSCs) (12, 13), osteoclasts (14), endothelial cells (15), macrophages (16), T regulatory cells (17–19), dendritic cells (20), plasmacytoid DCs (pDCs) (21), myeloid-derived suppressor cells (22), and mesenchymal cells (13, 23). These accessory cells secrete various cytokines including interleukin-6 (IL-6) (24), tumor growth factor β (TGFβ) (25, 26), macrophage inflammatory protein-1α (MIP-1α) (27), insulin-like growth factor (28), vascular endothelial growth factor (29), hepatocyte growth factor (30), B cell activating factor (BAFF) (31, 32), and a proliferation-inducing ligand (APRIL) (31, 33), which further maintain an MM-supporting or immunosuppressive BM microenvironment (34). For example, the key myeloma growth factor IL-6 and the critical immune inhibitory factor TGFβ are detected at high levels in the BM of MM patients. The interplay of these two cytokines may affect generation of Th17 cells both directly or *via* other pro-inflammatory cytokines, and thereby downregulate antitumor immune responses (35). Increased Th17 cells and decreased regulatory T cells (Tregs) with less immune suppression is noted in MM patients with long-term survival (36). Since Tregs can inhibit function of antigen-presenting cells and effector T cells (37), increased Treg number allows MM cells to escape from immune surveillance. In fact, immune-suppressive Treg markers Foxp3 and CTLA-4 are significantly upregulated in the BM aspirates of MM patients compared with normal donor controls (17), and increased Tregs are correlated with worse outcomes in MM (36, 38, 39). These studies indicate that molecular and cellular components suppress immune BM milieu, further enhancing MM progression.

Successful targeted anti-MM immunotherapies should both target MM cells and simultaneously restore antitumor activity of immune effector cells (40). Ideally, targets for effective immunotherapies should be selectively and strongly expressed on the surface of MM cells relative to normal cells. Compared with CD38 and SLAMF7, B cell maturation antigen (BCMA) demonstrates highly restricted expression on PCs but no other tissues, is, therefore, an excellent target for immunotherapy in MM (41, 42).

## BCMA is an Important Surface Protein Supporting the Survival of MM Cells

**Figure 1:**
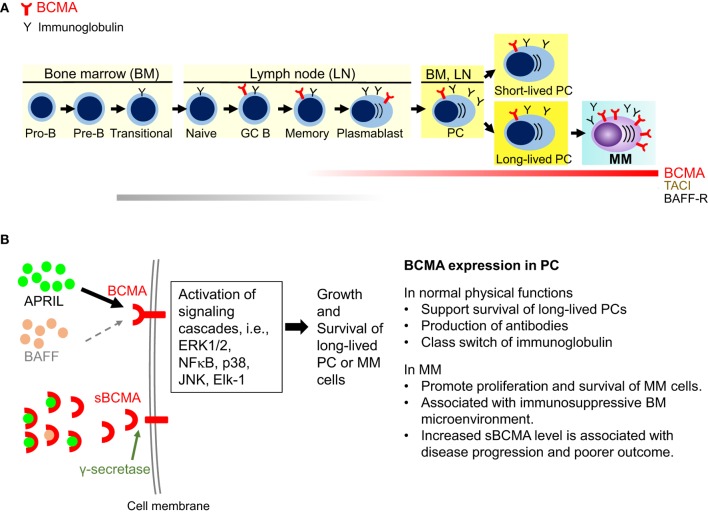
Biological significance of B cell maturation antigen (BCMA) in plasma cells (PCs). **(A)** BCMA is selectively induced during PC differentiation, associated with loss of BAFF-R. It is expressed on late-stage B-cells, short-lived proliferating plasmablasts, and long-lived PCs. BCMA does not maintain normal B-cell homeostasis but is required for the survival of long-lived PCs. In multiple myeloma (MM), expression of BCMA is significantly increased on malignant vs normal PCs. **(B)** A proliferation-inducing ligand (APRIL) and BAFF are two natural ligands for BCMA. Specifically, APRIL binds to BCMA with a significantly higher affinity than BAFF. Activation of BCMA supports growth and survival of PCs *via* activating MEK/ERK, AKT, NFκB, JNK, p38 kinase, and Elk-1. In MM cells, overexpression of BCMA or binding of APRIL to BCMA activates AKT, ERK1/2, and NFκB pathways and upregulate antiapoptotic proteins, i.e., Mcl-1, Bcl-2, Bcl-xL to protect MM cells from dexamethasone- and interleukin-6 deprivation induced apoptosis. Furthermore, BCMA upregulates genes associated with activation of osteoclast, adhesion, and angiogenesis/metastasis. Moreover, overexpressed BCMA can induce the expression immunosuppressive molecules such as PD-L1 in MM cells. Membrane BCMA can be cleaved by γ-secretase, resulting in reduced number of membrane-bound BCMA molecules and increased soluble BCMA. Soluble BCMA can bind to APRIL and BAFF, which may interfere downstream BCMA signaling cascades. TACI, transmembrane activator and calcium modulator and cyclophilin ligand interactor; GC, germinal center.

B cell maturation antigen, also termed tumor necrosis factor receptor superfamily member 17 (TNFRS17), is a type III transmembrane protein without a signal-peptide and containing cysteine-rich extracellular domains (43–45). Alignment of the human (44, 45) and murine BCMA protein sequences (43) revealed a conserved motif of six cysteines in the N-terminal part, which strongly suggests that the BCMA protein belongs to the tumor necrosis factor receptor (TNFR) superfamily. BCMA, along with two related TNFR superfamily B-cell activation factor receptor (BAFF-R) and transmembrane activator and calcium modulator and cyclophilin ligand interactor (TACI), critically regulate B cell proliferation and survival, as well as maturation and differentiation into PCs. These three functionally related receptors support long-term survival of B cells at different stages of development by binding to BAFF and/or APRIL (46–49), their cognate ligands. Specifically, BCMA is only induced in late memory B cells committed to the PC differentiation and is present on all PCs (46, 50, 51). Expression of BCMA is induced, while BAFF-R is decreased, during PC differentiation from B cells. Studies from BCMA-knockdown mice further indicate that BCMA is most important for long-lived PC survival but is dispensable for overall B cell homeostasis (50, 52). A recent study showed that an enzyme, γ-secretase can cleave membrane BCMA, leading to decreased in membrane form BCMA and formation of soluble form BCMA (sBCMA) (53) (Figure 1).

Earlier studies show that overexpression of BCMA in 293 cells activates the mitogen-activated protein kinase pathway, especially JNK and p38 kinase, the nuclear factors NFκB and Elk-1, without stimulation of BAFF or APRIL (54). BCMA expression is positively regulated by B-lymphocyte-induced maturation protein 1 (Blimp-1), a gene controlling proliferation of PCs (55). In KMS12 MM cell line, BCMA co-immunoprecipitates with interferon regulatory factor-4, a master transcription factor mediating survival of MM cells (56). Importantly, BCMA overexpression or APRIL binding to BCMA in MM cells significantly promotes MM cell growth and survival *in vivo* (33, 57). Conversely, BCMA knockdown blocks MM cell proliferation and viability *via* downregulation of cell cycle progression and antiapoptosis molecules. APRIL and BAFF, *via* binding to BCMA and TACI, further activate NFκB pathways and upregulate antiapoptotic proteins (Mcl-1, Bcl-2, Bcl-xL) to protect MM cells against dexamethasone- and serum deprivation-induced cell death (31, 58, 59) (Figure 1). These studies establish a pathophysiological role of BCMA and APRIL in MM.

## Rationale to Targeting BCMA in MM

B cell maturation antigen is exclusively expressed on the surface of plasmablasts and differentiated PCs, but not on memory B, naive B cells, CD34+ hematopoietic stem cells, and other normal tissue cells (41, 50, 51, 60–64). BCMA mRNA and protein are more highly expressed on malignant than normal PCs, as validated by multiple gene expression profiling (41, 42, 65, 66) and immunohistochemistry (IHC) studies (41). In the study by Carpenter et al. (41), cDNA copies of BCMA were detected by qPCR in several hematologic tissues including white blood cells, BM, lymph node, spleen, and tonsil. In normal tissues, low levels of BCMA cDNA copies were detected in the samples of testis, trachea and samples from gastrointestinal organs like duodenum, rectum, and stomach. When the expression was evaluated by IHC, BCMA protein expression was only detected on MM cells, lymphoid cells, or PCs from normal human organs such as duodenum, rectum, and stomach. However, BCMA protein expression was not detected on the other cell types in these organs (41). Another study examined BCMA expression on various blood cells and Hodgkin lymphoma cells using flow cytometry (63). BCMA expression was negative on naive and memory B cells, weak on founder B cells from germinal center (GC) and Reed–Sternberg cells, positive on GC B cells, but highly positive on plasmacytoid B cells. Based on these findings, BCMA protein is highly and specifically expressed on PCs, low levels of BCMA RNA detected in these normal organs would be due to existence of PCs.

Thus far, majorities of studies indicate that BCMA transcript, protein, and the serum BCMA level are significantly higher in MM cell lines and patient MM cells, when compared with normal donors. One recent study reported that median BCMA expression on patient MM cells was not to be higher compared to normal BMPC as shown in large patient cohorts (66). At protein levels, shedding of BCMA by γ-secretase controls PCs in the BM and may represent a potential biomarker for B-cell involvement in human autoimmune diseases (53). Significantly, sBCMA levels are increased in MM patients vs healthy individuals (67–69). Its level in patient serum is further correlated with disease status and prognosis. Furthermore, anti-BCMA antibodies are detected in MM patients in remission after donor lymphocyte infusion with graft-vs-tumor response, suggesting that antibody responses to cell-surface BCMA may directly contribute to tumor elimination (70). Moreover, low BCMA is detected in pDCs (42), which support survival and drug resistance of MM cells (21). In fact, pDCs are the only cell type other than PCs with detectable BCMA at significantly lower levels (>1-log lower) compared to matched PCs (42). These data confirm BCMA as a very promising MM antigen for targeted immunotherapy.

Both BCMA ligands APRIL and BAFF, to a lesser extent, are critical BM factors supporting growth and survival of malignant PCs in MM (31, 62). The levels of both ligands are significantly increased in serum samples of MM patients vs normal controls (31, 71). APRIL, which does not bind to BAFF-R, preferably binds to BCMA with much higher affinity than BAFF (nM vs μM), whereas BAFF has an approximate 100-fold selectivity for binding to BAFF receptor (BAFF-R) over BCMA (72, 73). Coupled with the fact that APRIL also binds to TACI on PCs *via* interaction with CD138/syndecan-1, APRIL is more specific to PCs than BAFF (57, 74–76). Importantly, APRIL directly promotes MM cell growth and survival *in vivo*, since APRIL knockout mice injected with human MM cell lines live longer than wild-type mice under similar conditions (77). These results strongly supporting targeting BCMA for novel MM treatments.

As described below, GSK2857916, the first therapeutic anti-BCMA antibody–drug conjugates (ADCs) with multiple mechanisms of action against MM cells, used alone and with MM-protecting BM components, rapidly eliminates MM cells in two murine models and significantly prolongs survival of mice (42). These promising data further support clinical development of BCMA-targeted immunotherapies in MM.

## BCMA-Based Immunotherapies

**Figure 2:**
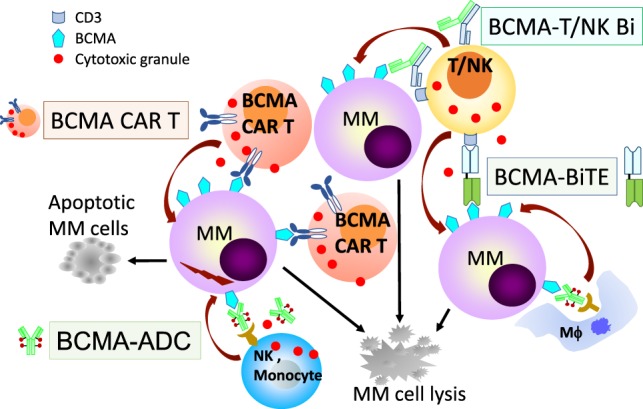
B cell maturation antigen (BCMA)-based immunotherapies with multiple mechanisms of action against MM cells. Various BCMA-based treatment modalities are under clinical development are listed in Table 1 and shown here. BCMA-NK Bi or Tri Ab, not shown here, can also specifically induce effector cell-mediated lysis of MM cells. ADC, antibody drug conjugate; Bi, bispecific full-length immunoglobulin; BiTE, bispecific T-cell engager; CAR T, chimeric antigen receptor T cell; MM, multiple myeloma cell; NK, natural killer cell; Mϕ, macrophage.

The development of novel agents targeting BCMA is ongoing rapidly, especially following impressive clinical responses in relapsed MM patients using the first chimeric antigen receptor (CAR) T cell therapy (78). Currently, there are multiple BCMA-based treatment modalities including: ADC, bispecific T-cell engager (BiTE), CAR T cell (CAR T), bispecific molecule, and bi/trispecific Abs (Figure 2) (summarized in Table 1), as well as cancer vaccines.

**Table 1:** List of Anti-B cell maturation antigen (BCMA) formats.

Therapeutic format	Compound (or name)	Company/sponsor	Characteristics	Clinical development	Reference
Antibody–drug conjugates	GSK2857916	GlaxoSmithKline	Humanized and afucosylated IgG1 mAb BCMA binding affinity: Kd of ~0.5 nM Anticancer drug: monomethyl auristatin F Linker: Maleimidocaproyl (non-cleavable)	Phase 1	(42, 79, 80)

	HDP-101	Heidelberg Pharma	Antigen-targeted amanitin-conjugates Humanized mAb Anticancer agent: Amanitin Linker: Maleimide (non-cleavable)	Preclinical	(81, 82)

	MEDI2228	MedImmune	Fully humanized antibody Anticancer drug: Pyrrolobenzodiazepine Linker: Protease-cleavable linker	Preclinical	(83)

Bispecific T-cell engager	BI 836909 (Amg420)/Amg701	Boehringer Ingelheim/Amgen	Bispecific single-chain variable fragment with hexahistidine tag Targeting CD3 and BCMA	Preclinical	(84, 85)

CAR T	Anti-BCMA chimeric antigen receptor (CAR)	National Cancer Institute	Transfection: γ-retroviral vector Extracellular domain: murine scFv Co-stimulation domain: CD28	Phase 1	(41, 78, 86)

	bb2121	Bluebird Bio Celgene	Transfection: Lentivirus vector Extracellular domain: Murine scFv Co-stimulation domain: 4-1BB	Phase 1	(87)

	LCAR-B38M	Nanjing Legend Biotech	Transfection: lentivirus vector Extracellular domain: Bispecific variable fragments of llama heavy-chain antibodies Co-stimulation domain: 4-1BB	Phase 1	(88, 89)

	CART-BCMA	Novartis	Transfection: Lentivirus vector Extracellular domain: fully human scFv Co-stimulation domain: 4-1BB	Phase 1	(90, 91)

	KITE-585	Kite Pharma	Transfection: lentivirus vector Extracellular domain: fully human scFv Co-stimulation domain: CD28	Preclinical	(92)

	BCMA CAR	Pfizer Cellectis SA	Transfection: lentivirus vector Extracellular domain: fully human scFv Co-stimulation domain: 4-1BB Inactivation of the T cell receptor alpha chain Contained an intra-CAR rituximab-recognition domain to deplete CAR T cells	Preclinical	(93)

	P-BCMA-101	Poseida Therapeutics	*In vitro* transcribed mRNA and plasmid DNA, no viral transfection Extracellular domain: human fibronectin type III domain Contain a safety switch	Preclinical	(94–96)

	FHVH74-CD828Z FHVH32-CD828Z FHVH33-CD828Z FHVH93-CD828Z	Tenebrio	Antigen-recognition domains composed of single fully human FHVH without light chain variable region domain or linker Co-stimulation domain: 4-1BB or CD28	Preclinical	(97)

	Descartes-08	Cartesian Therapeutics	CD8+ anti-BCMA CAR T-cells modified transiently by mRNA transfection	Preclinical	(98)

	P-BCMA-ALLO1	Poseida Therapeutics	NextGEN™ (NG) CRISPR gene editing system to disrupt both TCR and MHCI expression Non-viral piggyBac™ (PB) DNA transposition technology to produce CAR-T cells with highly desirable stem cell memory T cell subset	Preclinical	(99)

	EGFRt/BCMA-41BBz	Juno	Transfection: lentivirus Extracellular domain: fully human scFv Co-stimulation domain: 4-1BB Suicidal gene: EGFRt	Phase 1 (recruiting)	(89)

Bispecific molecule	BCMA/CD3 bispecific	Pfizer Alexo Therapeutics Kodiak Sciences	Fully-human IgG CD3 bispecific molecule with IgG2A backbone BCMA binding affinity: Kd 20 pM CD3 binding affinity: Kd ~40 nM	Preclinical	(100)

Bispecific antibody	EM801	EngMab AG Celgene	Two-arm IgG1-based human antibody One CD3 and two BCMA binding sites BCMA-binding affinity: Kd of 10 nM CD3-binding affinity: Kd of 70 nM	Preclinical	(66)

	^a^BCMA-TCB2/EM901	Celgene	Two-arm IgG1-based human antibody One CD3 and two BCMA-binding sites	Preclinical^a^	(101)

	Ab-957	Janssen	BCMAxCD3 bispecific antibody Ec50: BCMA + cell: 0.06–0.45 nM T-cell activation: 0.1–0.28 nM	Preclinical	(102)

	AFM26	Affimed	Targeting CD16A (NK cells) and BCMA NK-cell binding affinity: Kd of 1.2 nM	Preclinical	(103, 104)

	TNB383B/TNB-384B	TeneoBio	Targeting BCMA and CD3 Very low or absence of cytokine release after TNB-383B treatment	Preclinical	(105)

Trispecific antibody	Anti-CD16A/BCMA/CD200 antibody	Affimed	Trispecific antibody format: CD16A/BCMA/CD200 Bivalent binding to CD16A Monovalent binding to both BCMA and CD200	Preclinical	(106)

*Every effort has been made to obtain reliable data from multiple sources including http://clinicaltrials.gov/, companies, and other web sites, but accuracy cannot be guaranteed*.

*^a^Most recently, the BCMAxCD3 TCB CC-93269 (EM901) has entered clinical phase I testing (NCT03486067)*.

## Antibody–Drug Conjugates

Antibody–drug conjugate, one of the fastest growing class of cancer therapeutics, is composed of recombinant mAbs covalently bound to cytotoxic chemicals (payload) *via* synthetic chemical linkers (107). The mAbs first identify and bind to the antigen on the surface of tumor cells, and then is absorbed or internalized, together with the payload. After the ADC is internalized, the cytotoxic chemicals are released in the lysosomes and transported to cytosol to kill the tumor cells.

### GSK2857916

GSK285791 is a humanized and IgG1 mAb with high affinity to BCMA (Kd of ~0.5 nM) (42), which uses non-cleavable linker, maleimidocaproyl (mc), and a new class of antimitotic agents, monomethyl auristatin F, as payload. This structure is characterized by high stability and high antitumor potency, with low by-stander toxicity.

GSK2857916 binds to all CD138+ and BCMA+ MM cell lines and patient MM cells. MM cell proliferation is inhibited *via* G2/M arrest in a dose-dependent manner, and apoptosis is induced by activation of caspase 3/7 and 8. There are minimal effects on surrounding BCMA-negative normal cells. GSK2857916 also triggers ADCC and antibody-dependent cellular-mediated phagocytosis against patient MM cells. The cytotoxicity against MM cells is further enhanced when GSK2857916 is combined with lenalidomide *via* effector-dependent and -independent manners. Most importantly, in both disseminated and subcutaneous human MM xenograft models in mice, GSK2857916 rapidly eliminates MM cells and generated little toxicity in mice treated with continuous dosing for nine times at 4 mg/kg, with tumor-free survival up to 3.5 months in mice (42).

GSK2857916 was evaluated in a phase 1 study of patients with relapsed and refractory multiple myeloma (RRMM), including dose-escalating and expansion parts (79, 80). GSK2857916 monotherapy has demonstrated a 60% response rate and a median progression-free survival of 7.9 months in a group of hard to treat and heavily pretreated RRMM (80). It has recently been awarded Breakthrough Therapy designation from FDA and received PRIME designation from the European Medicines Agency (EMA).

### HDP-101

HDP-1, an antibody-targeted amanitin conjugate, is an anti-BCMA ADC with a novel payload amanitin, which binds to the RNA polymerase II in eukaryotic cells and inhibits cellular transcription at very low concentrations (108). HDP-1 was synthesized with the conjugation of maleimide-amanitin compounds and engineered cysteine residues in the heavy chain of the humanized anti-BCMA Thiomab (109, 110).

HDP-101 demonstrated potent *in vitro* cytotoxicity against BCMA-expressing MM cell lines at picomolar range, without effects on BCMA-negative cells. Significant tumor regression including complete remission was observed in the mouse xenograft model in a dose-dependent manner. The tolerability and therapeutic index were good after a series of HDP-101 administrations at different concentrations in Cynomolgus monkeys. Mild-to-moderate elevation of liver enzymes and lactic dehydrogenase were noted, but these abnormalities were transient. HDP-101 has a long half-life in serum (about 12 days) (81, 82).

### MEDI2228

The structure of MEDI2228 includes a fully human antibody site-specifically conjugated to a pyrrolobenzodiazepine dimer *via* a protease-cleavable linker. This ADC is rapidly internalized into MM cells and trafficked to lysosomes.

MEDI2228 was highly active in 8 of 10 MM cell lines (IC50 range 6 to 210 ng/mL) including cell lines regardless of BCMA levels (83). MEDI2228 was also active in the presence of BMSCs. A single injection of MEDI2228 induced human MM xenograft regression in mice at very low doses (0.1 mg/kg). MEDI2228 was characterized by weak binding capacity to recombinant monomeric human BCMA, but strong binding to membrane-bound BCMA. It kills an average of 95% of tumor cells in the presence of sBCMA at levels up to 720 ng/mL, without impact on IC50. Clinical trials of this new anti-BCMA will be starting in mid-2018.

## Bispecific T-Cell Engager

Bispecific T-cell engager is a single-chain variable fragment (scFv), composed of two linked mAbs (bispecific antibodies) targeting mainly CD3 on the surface of T-cells and tumor-associated antigens. This unique structure allows BiTE to engage T-cells with tumor cells (111). After the binding, antitumor cytotoxicity and cytokine production of T cells are activated, and the formation of cytolytic immunological synapses are induced (112, 113). BiTE is also characterized by its small size (55 kDa), which makes it a highly potent and efficacious molecule to against cancer (114). However, the small size of BiTE is unstable due to short serum half-life, thus continuous infusion is required.

### BI 836909

BI 836909 is the first bispecific scFv with two linked scFvs in MM (84). The scFv targeting BCMA is positioned in N-terminal, and the scFv targeting CD3ε is in C-terminal, followed by a hexa-histidine (His6-tag). BI 836909 simultaneously bind to CD3+ T cell and BCMA-expressing MM cells. This makes a cross-link between both cells to induce formation of cytolytic synapse, ultimately leading to activation of T cells and lysis of BCMA+ MM cells. These cytotoxic activities were not observed in BCMA-negative cells. When cocultured with BM stromal cells, BI 836909 retains potent anti-MM activity. Additionally, soluble APRIL and BCMA have only a mild effect on the anti-MM activity of BI 836909.

In mouse xenograft studies, BI 836909 led to tumor shrinkage in a subcutaneous NCI-H929 xenograft model and prolonged survival in an orthotopic L-363 xenograft model. In a cynomolgus monkey study, administration of BI 836909 resulted in significant depletion of BCMA + PCs in the BM of monkeys (84).

A half-life extended anti-BCMA BiTE base on BI 836909 was recently reported to be effective *in vitro* and *in vivo* and is suitable for once-weekly dosing in MM patients (85).

## CAR T Cell Therapy

Adoptive transfer of T cells genetically modified to recognize tumor-associated antigens is a promising cancer treatment (115). By using techniques of genetic modification, T cells can express CAR, which are fusion proteins that have an antigen recognition region, usually scFv derived from antibody on the surface, and a costimulation domain in the cell. Unlike T cell receptor modified T cells, CAR T cells are not restricted by major histocompatibility complex (40).

In MM, several anti-BCMA CAR T cell therapies have shown impressive clinical activities (some reaching 90–100%) with more are developed and under preclinical and/or clinical investigations (see Table 1).

## Other Trials of Anti-BCMA CAR-T Therapy

The combined infusion of CD19 and BCMA-specific CAR T Cells for RRMM was investigated in an early phase study (NCT 03196414) (116). The cells contained respective anti-BCMA or anti-CD19 scFv transduced by lentivirus, OX40 and CD28 costimulatory moiety, and CD3z T-cell activation domain. Clinical efficacy was evaluated in five patients monitored for more than 4 weeks, and showed that ORR was 100%, including 1 sCR, 1 VGRP, 2 PR, and 1 SD (116).

## Anti-BCMA CD3 BI- or Trispecific Molecules

### A Fully Human IgG CD3 Bispecific Molecule Targeting BCMA

This fully-human IgG bispecific molecule is characterized by its long half-life (about 3 days in mice) (100). The molecule utilizes hinge mutation technology to pair anti-BCMA and anti-CD3 targeting arms and places them in an IgG2A backbone. The anti-MM cytotoxicity was observed in MM patient samples at very low concentration (EC50 = 0.093 ± 0.1 nM), lower than ADC. This molecule also effectively depleted low BCMA-expressing normal plasma B cells. The evolution of toxicity in cynomologus monkeys model showed favorable safety profile.

### EM801

EM801 is asymmetric two-arm IgG1-based human antibody with two binding sites for BCMA and 1 binding site for CD3 (66). EM801 promotes activation of CD4+ and CD8+ T-cells accompanied with release of IFN-γ, granzyme B, and perforin, and CD3+ T cell-dependent killing of MM cell lines. EM801 also induced significant cell death in malignant PCs by autologous T cells in BM samples of previously untreated and RRMM patients at very low concentrations (from 10 pM to 30 nM).

### BCMA-TCB2

B cell maturation antigen-TCB2 is a bispecific antibody, which shares similar structure of EM801, but with higher affinity to BCMA (101). BCMA-TCB2 induces lysis of MM cells, activation of T cells, and natural emergence of the checkpoint inhibitor PD-1 on T cells at very low concentration. Combination of BCMA-TCB2 with lenalidomide or daratumumab significantly enhanced antimyeloma efficacy. NK cells were also activated after BCMA-TCB2 treatment.

### Ab-957

Ab-957 is bispecific IgG-like Ab generated by Genmab DuoBody^®^ technology to target CD3 on T cells and BCMA on MM cells (102). Preclinical studies also show that Ab-957 potently induces specific cytotoxicity of BCMA + MM cells *in vitro* and *in vivo*, with a concomitant activation of T cells at very low concentration.

### AFM26

AFM26 is a bispecific antibody, which targets BCMA on MM cells and CD16A on NK cells (103, 104). AFM26 induces potent NK-cell-medicated cytotoxicity in BCMA+ MM, even when BCMA expression of BCMA was low. AFM26 does not induce NK-cell depletion. It shows similar anti-MM activity, but less inflammatory cytokine secretion, than BiTEs.

### TNB383B and TNB-384B

TNB383B and TNB-384B are bispecific antibodies targeting BCMA on MM cells and CD3 on T cells, which are generated based on the basis of *in silico* analysis of heavy chain only/fixed light chain antibody sequences (105). Both Abs showed significant anti-MM cytotoxicity at very low concentration (nano- or pico-molar) and eradicated MM cell growth in mice. In addition, markedly reduced or absence of cytokine release is observed after TNB-383B treatment.

### Anti-CD16A/BCMA/CD200 Antibody

This trispecific antibody is characterized by bivalent binding to CD16A on NK cells and monovalent binding to BCMA and CD200 on MM cells (106). This dual-targeting structure may increase selectivity of MM cells coexpressing both antigens and improve safety.

## Therapeutic Agents Targeting APRIL

Therapeutic agents blocking APRIL/BCMA are under investigated as well. A novel mouse anti-human APRIL antibody hAPRIL01A (01A) inhibits the binding of APRIL to BCMA and TACI (117). Importantly, 01A inhibited APRIL- and osteoclast-induced proliferation of MM cells and further induced apoptosis of MM cells in cocultures (33). 01A also enhances the cytotoxicity mediated by IMiDs and PI in the cocultures of MM cells with BCMA-negative BM accessory cells and effector cells. Furthermore, APRIL induces expression of genes involved in immunosuppression, such as PD-L1, TGF-β, and IL-10, are decreased in MM cells following 01A treatment (33). The early phase clinical trial of BION-1301, a fully humanized 01A mAb, is ongoing (118).

## Perspectives and Conclusion

Since its discovery in 1992, accumulating evidence has demonstrated that BCMA is a promising target for immunotherapy in MM (Table 2). CAR T therapy first demonstrated promising clinical efficacy in several phase 1 clinical trials in which high response rates are seen in heavily pretreated RRMM patients. GSK2857916, the first therapeutic BCMA-ADC, also shows impressive clinical efficacy and acceptable safety profile in RRMM resistant to multiple lines of current anti-MM treatments (Table 3). Similar efficacy in clinical trials can be anticipated for other anti-BCMA formats demonstrating highly selective anti-MM activity in preclinical studies.

**Table 2:** Important milestone of anti-B cell maturation antigen (BCMA) immunotherapy for MM.

Years	Major findings	Reference
1992	BCMA gene was first found, which was located on chromosome band 16p13.1 in a human malignant T-cell lymphoma	(45)

1994	The structure of BCMA was investigated. BCMA is expressed in mature B cells	(44)

1998	BCMA gene was identified as a new member of the tumor necrosis factor receptor superfamily	(43)

2000	BCMA is the receptors of BAFF and a proliferation-inducing ligand	(47–49)
	BCMA is expressed both on the surface and in an intracellular perinuclear structure of myeloma cell	(54)
	Overexpressed BCMA can activate the MAPK pathway and the nuclear factors NF-κB and Elk-1	

2001	In mouse model studies, knock out of BCMA had no significant impact on the life span of B cell. The humoral responses and memory responses remained intact	(52)

2002	Gene array study identified expression of BAFF, TACI, and BCMA in myeloma cells	(65)

2004	BCMA is necessary for the survival of long-lived bone marrow plasma cells (PCs)	(50)
	BCMA is highly expressed in malignant PCs	(62)

2007	Anti-BCMA MoAb and antibody–drug conjugate (ADC) were synthesized	(119)
	Preclinical study showed antimyeloma activity in myeloma cell lines	

2013	The first anti-BCMA chimeric antigen receptor (CAR) T was synthesized (by NCI)	(41)
	This study confirmed BCMA to be exclusively expressed on malignant PCs	

2014	Anti-BCMA ADC (GSK2857916) showed antimyeloma activity by induction of apoptosis and ADCC	(42)

2016	First phase 1 clinical trial of anti-BCMA CAR T therapy reported	(78)
	First phase 1 clinical trial of anti-BCMA ADC reported (GSK2857916)	(79)
	Promising results of several phase 1 clinical trials	(78, 79, 90)

2017	High complete response rates to anti-BCMA CAR T therapy in relapsed and refractory multiple myeloma patients	(87, 88)

**Table 3:** Summary of phase 1 clinical trials of anti-B cell maturation antigen (BCMA) agents.

	Name	Enrollment criteria	No.	Prior treatment	Protocol	Results and efficacy	Adverse event (AE)
Antibody–drug conjugate	GSK2857916 (79, 80)	RR MM or other hematologic malignancies expressing BCMA	Dose-escalating part 24 (multiple myeloma)	83%, ≥4 prior lines (alkylators, PIs, IMiDs, ±stem cell transplantation)	IV infusion for 1 h ever 3 weeks 8 dose levels 0.03, 0.06, 0.12, 0.24, 0.48, 0.96, 1.92, 3.4 mg/kg	1 MR at 0.24 mg/kg 1 VGPR, 3 PR, and 1 MR at doses ≥0.96 mg/kg Clinical benefit rate: 25%	Overall: 23/24 (96%), nausea (42%), fatigue (38%), anemia (29%), chills (29%), pyrexia (29%), thrombocytopenia (29%), dry eye (21%), hypercalcemia (21%) Gr 3/4 SE (>10%): thrombocytopenia, anemia, and neutropenia Severe AEs: 8 (in 6 patients), including 1 unresolved limbal stem cell dysfunction Dose reduction: 4 patients IRR: 7/24 (29%) DLT (−)

			Expansion part 35	50%, ≥5 prior lines (range 1–10) All received PI and IMIDs 97% refractory to PI 91% refractory to IMiDs 40% received DARA (37% refractory) 89% refractory to PI and IMiDs	IV infusion for 1 h ever 3 weeks	1 sCR, 2 CR, 15 VGPR, and 3 PR PFS: 7.9 months	All patients had at least one AE Corneal events (63%), thrombocytopenia/platelet count decreased (57%), anemia (29%), AST increased (29%), and cough (26%) Gr 3/4 AEs (≥10%): thrombocytopenia (34%) and anemia (14%) Serious AEs were reported in 40% (14/35) of pts IRRs: 8 (2 Gr 1, 3 Gr 2, 3 Gr 3)

Chimeric antigen receptor (CAR) T	Anti-BCMA CAR (78)	RRMM BCMA expression by either IHC or FCM	12	Median of 7 prior lines (range 3–13)	Cy (300 mg/m^2^) 3 doses and Flu (30 mg/m^2^) 3 doses Followed by dose escalation of CAR T from (0.3, 1, 3, 9) × 10^6^ cells/kg	1 sCR, 2 VGPR, 1 PR, 8SD	Gr 3/4 AE: lymphopenia (100%), leukopenia (100%), neutropenia (100%), anemia (50%), thrombocytopenia (50%)

	bb2121 (120)	RRMM 50% BCMA expression on plasma cells	21 (18 evaluable for response)	Median of 7 prior lines (range 3–14) All received auto-HSCT 71% exposed to Bort/Len/Car/Pom/Dara 29% with penta-refractory	Lymphodepletion: flu (30 mg/m^2^)/Cy (300 mg/m^2^) daily for 3 days Followed by 1 infusion of bb2121 3 + 3 design with planned dose levels of 50, 150, 450, 800, and 1,200 × 10^6^ CAR T cells	Median follow-up after Bb2121 infusion: 15.4 weeks 1. ORR:89% (16/18) 2. ORR:100% (15/15, with 150 × 10^6^ or more CAR T cells) 4CR, 7 VGPR, 4PR (4 MRD-) 3. MTD: 80 × 10^7^ CAR + T-cells	CRS: 15/21 (71%), grade3 (*n* = 2) Gr 3/4 AE: lymphodepletion, hyponatremia (*n* = 4), CRS (*n* = 2), URI (*n* = 2), and syncope (*n* = 2) No DLT 1 death (cardiopulmonary arrest) more than 4 months after bb2121 infusion in a patient with an extensive cardiac history (disease status: sCR)

	LCAR-B38M (88, 121)	RRMM	19 (evaluable)	≥3 prior regimens	Median infusion cells: 4.7 (0.6–7.0) × 10^6^/kg, 3 infusions in 6 days	1. ORR:100%, with 14 sCR, 4 VRPR, 1 PR	CRS:14 (74%), Gr 3/4 (*n* = 2) No neurologic AEs

		RRMM, with extramedullary involvement	5 (2 with EMD)	All relapsed after classical chemotherapy, IMiDs, and PIs 3 with prior auto-HSCT	Pre-CAR-T treatment: fludarabine (25 mg/m^2^) and cyclophosphamide (250 mg/m^2^) daily for 3 days (d-5–d-3) 0.62 × 10^6^/kg (median) CAR-T cells for 3 days (d0, d2, and d6)	1. 1 CR, 1VGPR, 3 PR	Most common AEs: CRS DLT (−) TRM (−)

	CART-BCMA (90)	RRMM	33 consented 28 eligible 21 infused	Median 7 prior lines of therapy (range 3–11) 100% PI and IMIDs refractory 67% Dara refractory 95% had high-risk cytogenetics 67% del17p or TP53 mutation 29% extramedullary disease	3 split-dose infusions of CAR T cells (10, 30, 60%) 3 cohorts 1–5 × 10^8^ CART cells (*n* = 9) Cy 1,500 m g/m^2^ + 1–5 × 10^7^ CART cells (*n* = 5) Cy 1,500 mg/m^2^ + 1–5 × 10^8^ CART cells (*n* = 7)	18 (86%) received full planned dose, and 3 received 40% of dose Efficacy Cohort 1:1 sCR, 2 VGPR, 1 PR, 2 MR Cohort 2:1 PR, 1 MR Cohort 3:1 CR, 3 PR, 1 MR CAR T cell expansion By qPCR: 100% By FCM:90%	Cohort 1 data Grade 3/4 SE: hypophosphatemia (*n* = 3), hypocalcemia (*n* = 2), anemia, neutropenia, lymphopenia, thrombocytopenia, hypofibrinogenemia, fatigue, pneumonia, UTI, elevated ALP and AST, hypokalemia, hypertension, and pleural effusion CRS Cohort 1:8 (3 grade 3/4, with 4 receiving tocilizumab) Cohorts 2/3:9 (3 grade 3, none requiring tocilizumab) Neurotoxicity Cohort 1; 2 (grade 4 encephalopathy) Cohorts 2/3:1 (grade 2 confusion/aphasia) DLT (−)

*ALP, alkaline phosphatase; AST, aspartate aminotransferase; auto-HSCT, autologous hematopoietic stem cell transplantation; Bort, bortezomib; Car, carfilzomib; CRS, cytokine releasing syndrome; Cy, cyclophosphamide; Dara, daratumumab; DOR, duration of response; DLT, dose-limiting toxicity; EMD, extramedullary disease; Flu, fludarabine; FCM, flow cytometry; Gr, grade, IHC, immunohistochemistry; IMiD, immunomodulatory drug; IRR, infusion-related reaction; Len, lenalidomide; MoAb, monoclonal antibody; MR, minimal response; MRD, minimal residual disease; MTD, maximal tolerated dose; ORR, overall response rate; PCR, polymerase chain reaction; PD, progressive disease; PI, proteasome inhibitor; Pom, pomalidomide; PR, partial response; PRES, posterior reversible encephalopathy syndrome; RRMM, relapsed and refractory multiple myeloma; sCR, stringent complete response; SD, stable disease; URI, upper airway infection; UTI, urinary tract infection; VGPR, very good partial response*.

Ongoing efforts are attempting to make BCMA CAR T therapy more potent, safe, and affordable for patients. To improve clinical efficacy, novel CAR T therapies are being developed to overcome relapse due to reduced tumor antigen, including modification of T cells with two distinct CAR molecules with two different binding domains, or one CAR molecule with two different binding domains in tandem (122–124). To reduce toxicities of conditioning chemotherapy, possible approaches include usage of less toxic conditioning chemotherapy, treating earlier in the disease course with less tumor burden, and improved supportive care (125). For prediction of severe cytokine releasing syndrome (CRS), several inflammation cytokines (especially IL-6) have been evaluated, and models have been established (125, 126). For the treatment of CRS, cytokine-directed therapy with anti-IL6 receptor inhibitor tocilizumab can abrogate toxicities (127, 128). Other strategies to reduce side effects include modification of CAR structure, such as incorporation of suicide genes into the engineered T cells (129–131); adding an inhibitory CAR on engineered T cells to reduce off-target immune response (132); or usage of a small molecule system to control CARs (133, 134). More cost-effective, time-saving, and more accessible CAR T cell therapies are being developed, including allogeneic CAR T cells or CAR T cells utilizing novel manufacturing processes (93, 94).

For BCMA ADC, the first clinical trial has demonstrated efficacy and safety. ADC delivering highly toxic chemicals into the tumor cells is a highly selective therapy, which is critical since, the conjugated toxic chemicals are extremely deadly. Currently, several novel promising payloads are under development, including α-amanitin, tubulysins, hizoxin, or spliceostatins (135–137). To improve penetration, novel ADC formats such as non-IgG scaffolds or non-internalizing mAb scaffolds, may be applied to anti-BCMA ADC (138). Besides modification of ADC structure, combinations of ADC with other antitumor agents with different mechanisms of action are also under further investigation. Given that immune checkpoint inhibitors have clinical efficacy in several cancers, studies evaluating the clinical efficacy of combining immune checkpoint inhibitors with BCMA ADC are also warranted in MM (139).

Bispecific T-cell engagers are currently evaluated in preclinical studies. These anti-BCMA agents with excellent anti-MM effect will soon be investigated in clinical trials. Unlike CAR T cell therapy, BiTEs have a relatively short serum half-life and may not stimulate persistent immunity against cancer cells (140). Because it is difficult to maintain serum levels with bolus or intermittent infusion, continuous intravenous infusion may be needed (141). Importantly, long half-life molecules of BCMA BiTEs have been generated (85) and are currently being tested in a clinical trial. As CRS and neurotoxicity are also observed after BiTE treatment, close monitoring and adequate management for these side effects is very important (142). BCMA BiTEs mainly mediate their anti-MM effect by recruiting nearby cytotoxic T-cells to MM cells. However, the function of T cells is severely impaired in heavily pretreated MM patients (143, 144). To optimize BiTE anti-MM activity, studies are evaluating combination therapy with other anti-MM agents or immune checkpoint blockers.

Besides MM, anti-BCMA therapies may have therapeutic potential in other BCMA-expressing malignancies. For example, BAFF-R, BCMA, and TACI are all expressed on primary cells from patients with precursor B-cell acute lymphoblastic leukemia. Moreover, survival of leukemia cells is promoted by binding of BAFF and APRIL to their receptors, suggesting the therapeutic potential of targeting this signaling pathway (145). Other malignancies, such as Waldenstrom macroglobulinemia and glioblastoma/astrocytomas, also express BCMA on their cell surface (146, 147) and may benefit from these BCMA targeted therapies.

As BCMA is exclusively expressed on PCs, anti-BCMA treatment will reduce the number of long-lived PCs. Since long-lived PCs play a critical role in maintaining humoral immunity, the impact of anti-BCMA therapy on immune function needs to be carefully and serially evaluated. To address this issue, more clinical observation and correlative studies are warranted. Another potential complicating factor in anti-BCMA immunotherapy is high serum level of sBCMA, cleaved from BCMA by γ-secretase. In MM patients, high levels of sBCMA have been detected, especially in the setting of progressive disease (68). In preclinical studies, sBCMA slightly influenced the potency (shift in EC50 values) but not the maximal lysis mediated by BI 836909 (84). GSK2857916 still induced significant MM1S cell lysis in the presence of MM1S culture supernatant (42). On the other hand, sBCMA level is markedly decreased in patients after successful CAR T cell therapy (78). More clinical studies are needed to determine whether the level of sBCMA can potentially interfere with efficacy of anti-BCMA treatment. Inhibition of γ-secretase to reduce the formation of sBCMA and enhance the expression of BCMA on MM cells is another novel treatment approach.

In conclusion, BCMA-based immunotherapy is a promising in MM. It is anticipated that most of these anti-BCMA approaches, alone and in combinations with immune checkpoint inhibitors, and as well as cancer vaccines, will be evaluated in clinical studies and offer the promise of more selective, better tolerated, anti-MM therapy.

## Author Contributions

Y-TT and S-FC reviewed literature and designed and wrote this paper. Y-TT and KA critically reviewed and edited the paper.

## Conflict of Interest Statement

KA serves on advisory boards Celgene, Millennium, and Gilead Sciences and is a Scientific Founder of OncoPep and C4 Therapeutics. The other authors have no competing interests to declare.
